# Contrast-enhanced endoscopic ultrasound-guided fine-needle aspiration for a gastric submucosal tumor with surrounding hemorrhage

**DOI:** 10.1055/a-2257-3279

**Published:** 2024-02-15

**Authors:** Yuki Utakata, Takuji Iwashita, Shota Iwata, Akihiko Senju, Ryuichi Tezuka, Shinya Uemura, Masahito Shimizu

**Affiliations:** 1 476117 First Department of Internal Medicine, Gifu University Hospital, Gifu, Japan

Endoscopic ultrasound-guided fine-needle aspiration (EUS-FNA) represents a useful and less invasive procedure for procuring pathological specimens from lesions located either in proximity to or within the gastrointestinal wall 1 2 . Contrast-enhanced EUS (CE-EUS) is known to be advantageous for differential diagnosis in various types of tumors, as it provides real-time blood flow images 3 . In this report, we present a patient with a gastric submucosal tumor associated with hemorrhage, in whom CE-EUS-guided FNA (CE-EUS-FNA) had a pivotal role in precisely identifying the lesion and facilitating accurate FNA.

A 60-year-old man initially presented with abdominal pain. A computed tomography scan revealed the presence of a tumor on the gastric wall, with possible perigastric hemorrhage ( Fig. 1 ). Subsequently EUS-FNA was attempted on the gastric lesion, but EUS failed to detect the tumor because of the hemorrhage surrounding the stomach ( Fig. 2 ). Consequently, CE-EUS was performed, enabling the identification of a 20-mm tumor that was contiguous with the gastric wall ( Fig. 3 ). Subsequent CE-EUS-FNA using a 22-gauge fine-needle biopsy (FNB) needle was performed successfully and precisely ( Fig. 4 ; Video 1 ). No adverse events related to the procedure were recognized.

**Fig. 1:**
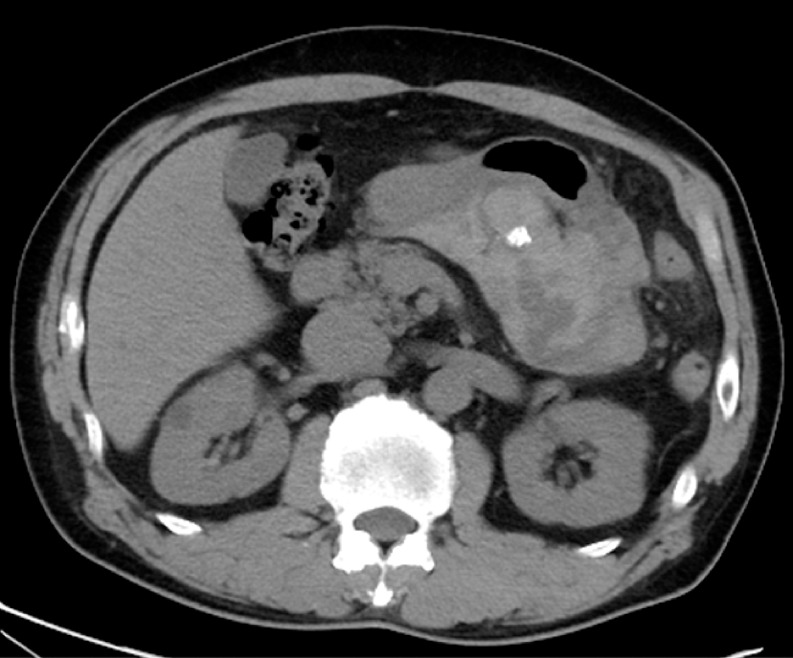
Computed tomography image showing a tumor connected with the gastric wall, with possible perigastric hemorrhage.

**Fig. 2:**
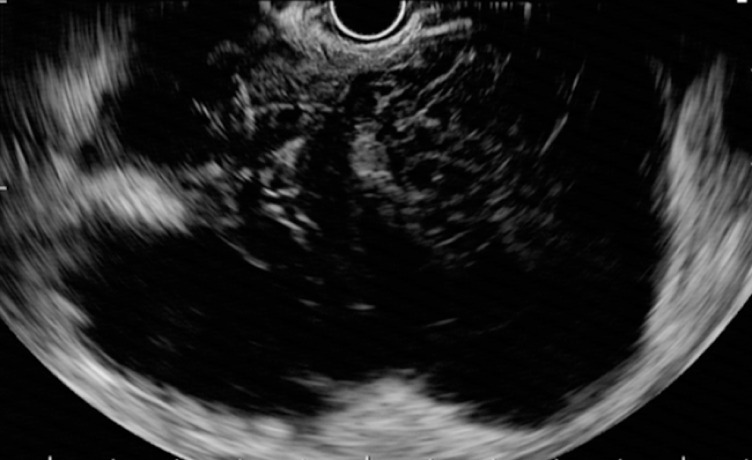
Endoscopic ultrasound image showing that the tumor connected with the gastric wall could not be detected because of hemorrhage around the stomach.

**Fig. 3:**
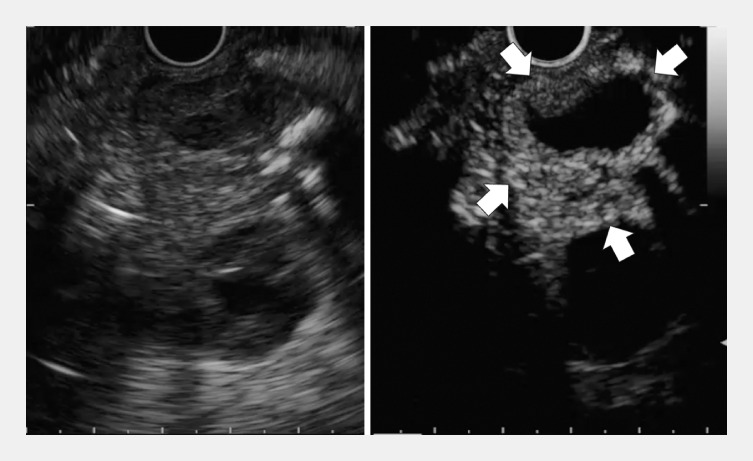
Contrast-enhanced endoscopic ultrasound images showing the 20-mm tumor (arrows), which was connected with the gastric wall.

**Fig. 4:**
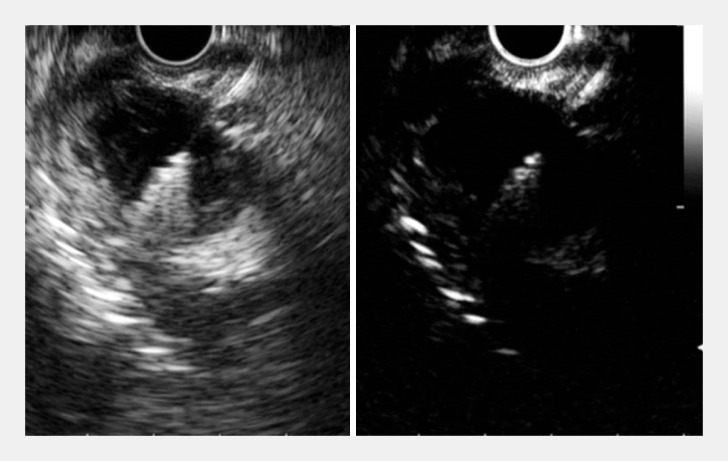
Contrast-enhanced endoscopic ultrasound images showing fine-needle biopsy (FNB), with a 22-gauge FNB needle, being successfully and accurately performed for the tumor.

Contrast-enhanced endoscopic ultrasound-guided fine needle aspiration was performed for a submucosal tumor with surrounding hemorrhage.Video 1

Pathological examination of the obtained specimens revealed tumor cells characterized by spindle-shaped nuclei with positivity for c-kit and CD34 ( Fig. 5 ). These findings were consistent with a diagnosis of gastrointestinal stromal tumor (GIST). Given the presence of intra-abdominal hemorrhage, surgical resection was undertaken following neoadjuvant therapy involving imatinib. The final pathological examination confirmed the diagnosis of a GIST.

**Fig. 5:**
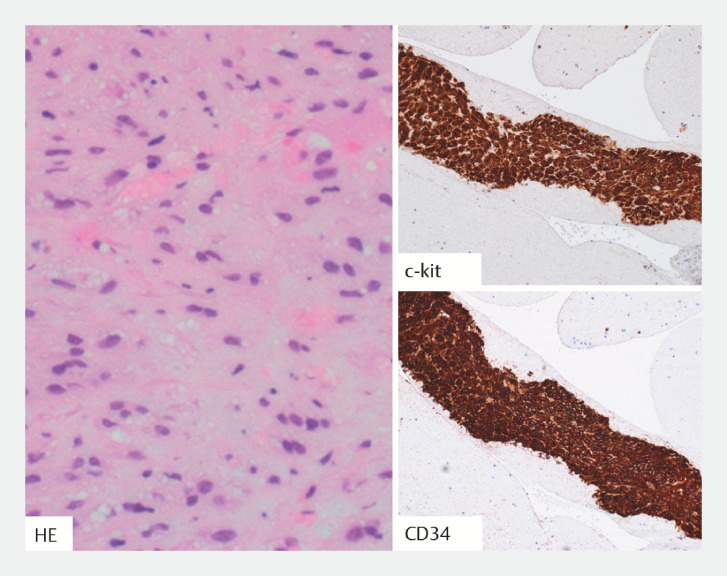
Pathological appearance of the specimen showing tumor cells with spindle-shaped nuclei on hematoxylin and eosin (H&E) staining, and positivity on immunohistochemistry with c-kit (+) and CD34 (+), consistent with a diagnosis of gastrointestinal stromal tumor (GIST).

Recent studies regarding CE-EUS-FNA for pancreatic lesions have not conclusively demonstrated its superior diagnostic capabilities over B-mode-based EUS-FNA 4 5 . In this case, however, CE-EUS enabled us to detect the tumor precisely and subsequently to perform precise needle puncture based on real-time perfusion imaging. CE-EUS-FNA could be useful in cases where lesion detection is interfered with by confounding factors such as hemorrhage.

Endoscopy_UCTN_Code_TTT_1AS_2AB

## Notes

Endoscopy E-Videos https://eref.thieme.de/e-videos
*E-Videos* is an open access online section of the journal *Endoscopy* , reporting on interesting cases and new techniques in gastroenterological endoscopy. All papers include a high-quality video and are published with a Creative Commons CC-BY license. Endoscopy E-Videos qualify for HINARI discounts and waivers and eligibility is automatically checked during the submission process. We grant 100% waivers to articles whose corresponding authors are based in Group A countries and 50% waivers to those who are based in Group B countries as classified by Research4Life (see: https://www.research4life.org/access/eligibility/ ). This section has its own submission website at https://mc.manuscriptcentral.com/e-videos .
